# Cerium molybdate-doped polyaniline nanoplatform for GSH-depletion-enhanced chemodynamic/NIR-II photothermal synergistic therapy via ferroptosis and immune activation

**DOI:** 10.1016/j.bioactmat.2026.06.035

**Published:** 2026-08-12

**Authors:** Yulin Kuang, Chen Lu, Bolan Yu, Lie Li, Fei Xiao, Xuyuan Chen, Junwei Wang, Liming Lei, Yijin Wu, Xinjian Yan, Shuoji Zhu, Min Wu, Nanbo Liu, Ruiyuan Liu, Ping Zhu

**Affiliations:** a School of Medicine, South China University of Technology, Guangzhou, Guangdong, 510006, China; b Guangdong Cardiovascular Institute, Guangdong Provincial People's Hospital (Guangdong Academy of Medical Sciences), Southern Medical University, Guangzhou, Guangdong, 510100, China; c Guangdong Provincial Key Laboratory of Pathogenesis, Targeted Prevention and Treatment of Heart Disease, Guangzhou Key Laboratory of Cardiac Pathogenesis and Prevention, Guangzhou, Guangdong, 510100, China; d Department of Anesthesiology, Second Affiliated Hospital of Guangzhou Medical University, Guangzhou, Guangdong, 510260, China; e Biomaterials Research Center, School of Biomedical Engineering, Southern Medical University, Guangzhou, 510515, China; f Comprehensive Medical Treatment Ward, Nanfang Hospital, Southern Medical University, Guangzhou, 510515, China

**Keywords:** Polyaniline, Near-infrared-II, Chemodynamic therapy, Ferroptosis, Antitumor immunity

## Abstract

Chemodynamic therapy (CDT) holds great promise for tumor treatment by catalyzing endogenous hydrogen peroxide (H_2_O_2_) into cytotoxic reactive oxygen species (ROS). However, its efficacy is severely compromised by glutathione (GSH)-mediated ROS scavenging and intrinsically slow reaction kinetics. To address these limitations, we developed cerium molybdate-doped polyaniline nanoparticles (MoCe@PANI NPs) to enable a synergistic combination of photothermal therapy (PTT) and CDT, thereby triggering immunogenic cell death (ICD) and enhancing antitumor immunity. Mechanistically, MoCe@PANI NPs deplete intracellular GSH and generate abundant ROS via Ce^4+^/Mo^5+^/Mo^6+^-mediated redox cycles, leading to mitochondrial dysfunction and amplified ferroptosis. Crucially, near-infrared II (NIR-II) laser irradiation induces localized hyperthermia, which simultaneously executes PTT and accelerates the Fenton-like reaction kinetics, further intensifying ferroptotic cell death. This combinatorial strategy effectively activates tumor-associated immune responses, achieving effective ablation of primary tumors and eliciting a systemic abscopal effect that suppresses untreated distant metastases. Collectively, this study presents a robust nanoplatform integrating chemodynamic and photothermal therapies for potent cancer immunotherapy.

## Highlights

• MoCe@PANI NPs synergize NIR-II photothermal therapy with GSH-depletion-enhanced chemodynamic therapy, where the photothermal effect significantly accelerates Fenton-like reaction kinetics for massive ROS generation.
• The synergistic therapy potently induces mitochondrial dysfunction and ferroptosis in tumor cells, characterized by GPX4 downregulation and extensive lipid peroxidation.
• This strategy triggers robust immunogenic cell death (ICD), remodels the immunosuppressive tumor microenvironment, and elicits a systemic abscopal effect to inhibit untreated distant metastasis.

## Graphical abstract

The mitochondrial-targeting MoCe@PANI nanoplatform was constructed to deplete GSH and boost NIR-II photothermal-enhanced Fenton kinetics, which could induce mitochondrial dysfunction, GPX4 downregulation, and lipid peroxidation, thereby facilitating high-efficiency ferroptosis-mediated photo-immunotherapy with abscopal effects.

**Figure:**
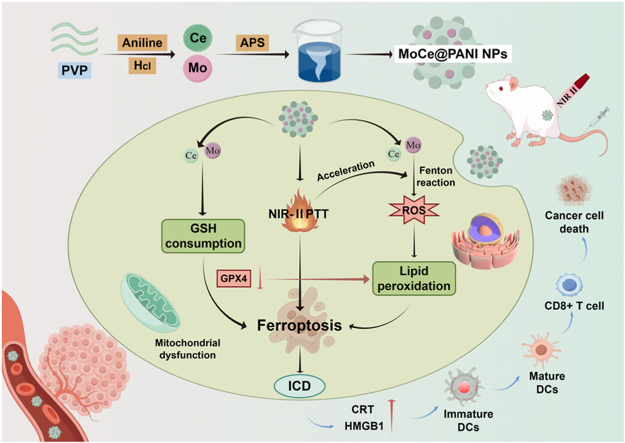

## 1 Introduction

Cancer presents a formidable global health challenge, necessitating the development of novel and innovative therapeutic strategies to effectively inhibit its progression and metastasis [1,2]. Conventional cancer treatments, including surgery, chemotherapy, and radiotherapy, are often limited by low specificity, severe systemic toxicity, drug resistance, and high recurrence rates, prompting the urgent exploration of advanced therapeutic modalities [[3], [4], [5]]. Among emerging strategies, chemodynamic therapy (CDT) has garnered significant attention as a promising tumor-specific approach [6,7]. CDT employs transition metal ions as catalysts to convert endogenous hydrogen peroxide (H_2_O_2_) within the tumor microenvironment (TME) into highly cytotoxic hydroxyl radicals (·OH) via Fenton or Fenton-like reactions, thereby inducing cancer cell apoptosis [[8], [9], [10]]. Notably, the reactive oxygen species (ROS) generated through this process can trigger extensive lipid peroxidation and cause irreversible mitochondrial damage, leading to ferroptosis—an iron-dependent form of regulated cell death that significantly enhances cancer cell lethality [[11], [12], [13]].

Despite considerable advancements in optimizing CDT agents, several intrinsic barriers impede their clinical translation [14]. A primary limitation is the elevated glutathione (GSH) levels in tumor tissues, which act as potent ROS scavengers, substantially diminishing therapeutic efficacy by disrupting oxidative stress balance [15,16]. Consequently, the depletion of intracellular GSH to disrupt redox homeostasis has been identified as a pivotal mechanism to augment CDT and ferroptosis [[17], [18], [19]]. To address this, recent studies have rationally moved beyond conventional Fe- or Mn-based catalysts toward multivalent metal pairs [20,21]. Specifically, cerium (Ce) and molybdenum (Mo) offer a superior synergistic electron transfer mechanism that overcomes the sluggish kinetics of traditional Fenton chemistry [22]. Ce^4+^ possesses a high redox potential (Ce^4+^/Ce^3+^ ≈ 1.72 V), enabling it to rapidly oxidize GSH and deplete this antioxidant defense while undergoing reduction [23,24]. Crucially, this facilitates the subsequent regeneration of active Mo^5+^/Mo^6+^ couples via electronic charge transfer within the doped polyaniline matrix [25,26]. This Ce-to-Mo electron relay not only sustains the catalytic cycle for continuous ·OH generation but also inherently amplifies the ferroptotic signal by disabling the GPX4-GSH axis, thereby providing a logical foundation superior to monometallic or simple oxide systems [[27], [28], [29], [30]].

Moreover, given that monotherapies often fail to achieve complete tumor eradication and prevent metastasis, combinatorial therapeutic approaches are gaining prominence for their ability to synergistically harness the advantages of individual treatments while mitigating their inherent limitations [31,32]. Photothermal therapy (PTT) has emerged as a highly attractive modality in oncology due to its minimally invasive nature, deep tissue penetration, and high spatial specificity [33,34]. Previous studies have demonstrated that the kinetics of ROS generation via the Fenton reaction are significantly accelerated at elevated temperatures, making PTT an ideal adjunct to enhance ferroptosis [35,36]. Beyond direct thermal ablation of tumor tissues, PTT also stimulates host antitumor immune responses by promoting immunogenic cell death (ICD) [37,38]. The damage-associated molecular patterns (DAMPs) released during ICD facilitate the maturation of dendritic cells (DCs) and the infiltration of cytotoxic T lymphocytes, orchestrating a robust systemic antitumor immune response [39]. Recent advances in nanomedicine further highlight that rational delivery platforms—including dendritic polymer-based systems to remodel tumor stroma [40] and bioinspired delivery vectors [41]—can significantly augment chemo-immunotherapy synergy, while immunometabolic interventions are being exploited to reprogram the immunosuppressive TME into an immune-activated state [42]. Although various nanoplatforms have been engineered for combined photothermal and chemodynamic therapy [43], their efficacy is often limited by insufficient penetration depth and heat shock responses [44,45]. Compared to the first near-infrared window (NIR-I, 700–900 nm), PTT operating within the second near-infrared window (NIR-II, 1000–1700 nm) offers superior tissue penetration, higher permissible exposure, and an enhanced capacity to trigger ICD, making it more suitable for deep-seated tumor ablation [[46], [47], [48], [49]].

**Scheme 1:**
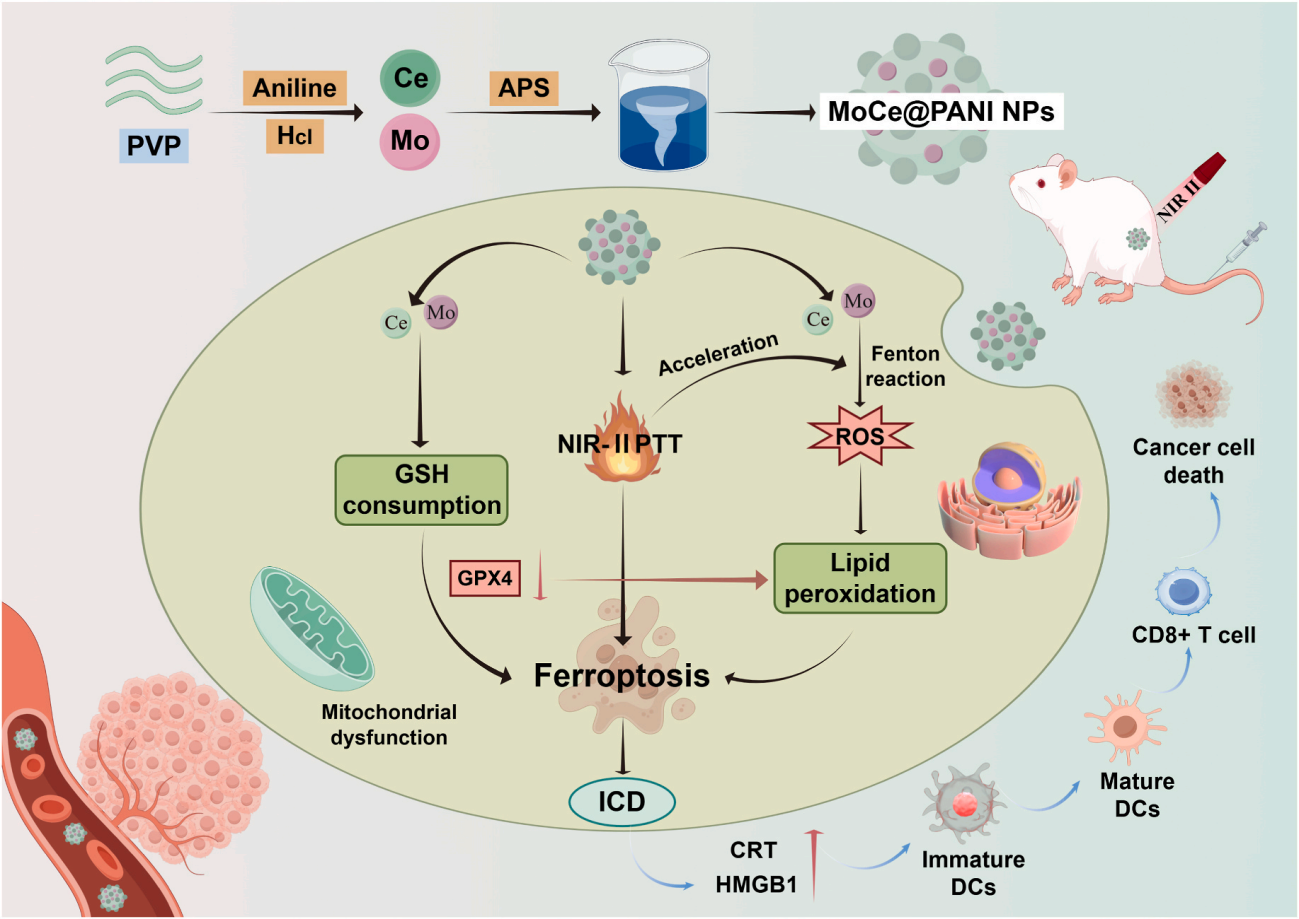
Schematic illustration of the synthesis of MoCe@PANI NPs and their mechanism for synergistic cancer therapy.

Herein, we engineered cerium molybdate-doped polyaniline nanoparticles (MoCe@PANI NPs) with intrinsic GSH-depletion capabilities to facilitate synergistic chemodynamic therapy and NIR-II PTT (see Scheme 1). The conductive PANI backbone facilitates the redox cycling of the Ce/Mo electron relay, effectively accelerating Fenton-like reaction kinetics under laser irradiation. The combination of MoCe@PANI NPs with NIR-II laser exposure induces intensified thermal damage, elevated intracellular ROS and lipid peroxidation levels, and severe mitochondrial impairment, culminating in pronounced ferroptosis. Furthermore, the ICD triggered by this synergistic therapy facilitates the release of DAMPs, thereby activating a robust immune response against tumor cells and inhibiting metastasis. *In vivo* experiments confirmed complete primary tumor ablation and the elicitation of a systemic antitumor immune response, effectively suppressing untreated distant tumors. This study presents a highly selective and efficacious antitumor modality that integrates GSH-depleted ferroptosis with NIR-II PTT, offering novel insights for the advancement of next-generation cancer treatment strategies.

## 2 Experimental procedure

### 2.1 Chemicals and materials

Aniline, ammonium persulfate (APS), ammonium molybdate tetrahydrate ((NH_4_)_6_Mo_7_O_24_·4H_2_O), cerium(IV) nitrate (CeN_4_O_12_), and 3,3′,5,5′-tetramethylbenzidine (TMB) were purchased from Aladdin Reagent Co., Ltd. Polyvinylpyrrolidone (PVP, Mw ≈ 40,000) was supplied by Energy Chemical. 5,5′-Dithiobis(2-nitrobenzoic acid) (DTNB) was obtained from Tokyo Chemical Industry.

Cell culture reagents including Dulbecco's Modified Eagle Medium (DMEM), fetal bovine serum (FBS), and penicillin-streptomycin were purchased from Gibco. Cell Counting Kit-8 (CCK-8) was obtained from Dojindo Laboratories. Calcein-AM/PI Detection Kit, 2′,7′-dichlorofluorescein diacetate (DCFH-DA), JC-1 Mitochondrial Membrane Potential Assay Kit, BODIPY 581/591 C11 probe, MitoSOX Red probe, GSH Assay Kit, and MDA Assay Kit were purchased from Beyotime Biotechnology Co., Ltd. Primary antibodies against GPX4 (ab125066) and GAPDH (ab181602) were obtained from Abcam. All flow cytometry antibodies (anti-CD86, CD80, CD4, CD8, CD69, F4/80, CD163, CD206, CD11b, and Gr-1) were purchased from BD Biosciences.

### 2.2 Synthesis of MoCe@PANI NPs

MoCe@PANI NPs were synthesized via a low-temperature one-pot oxidative polymerization strategy. Briefly, 500 mg of PVP was dissolved in 15 mL of DI water, followed by the sequential addition of 100 μL of aniline monomer and 100 μL of concentrated HCl under stirring. The mixture was pre-cooled in an ice-water bath (0–4 °C). Subsequently, pre-cooled aqueous solutions of ammonium molybdate tetrahydrate (100 mg in 5 mL) and cerium(IV) nitrate (100 mg in 5 mL) were added dropwise. Polymerization was initiated by slowly introducing 228 mg of ammonium persulfate (dissolved in 10 mL DI water and pre-cooled) and allowed to proceed for 6 h under continuous stirring until a dark green color developed. The resulting MoCe@PANI NPs were collected by centrifugation, washed repeatedly with DI water, and stored at 4 °C.

The synthesis procedures for MoCe@PANI NPs with 25 mg and 200 mg metal precursors, as well as free PANI (without Mo/Ce) and free MoCe (without PANI) control materials, are described in the Supplementary Information.

### 2.3 Characterization

The morphology and microstructure were observed using a Talos F200X transmission electron microscope. Hydrodynamic size and *zeta* potential were measured by dynamic light scattering (DLS) using a Zetasizer Nano ZS90. X-ray diffraction (XRD) patterns were recorded on a SmartLab SE diffractometer. Fourier transform infrared (FTIR) spectra were obtained using a Vertex 70 spectrometer with KBr pellets. X-ray photoelectron spectroscopy (XPS) analysis was performed on a K-Alpha spectrometer with monochromatic Al Kα radiation. The actual loading amounts of Mo and Ce were quantified by inductively coupled plasma mass spectrometry (ICP-MS, Agilent 7850). UV–vis–NIR absorption spectra were recorded on an Evolution 300 spectrophotometer (Thermo Scientific).

### 2.4 Photothermal performance and photothermal conversion efficiency

To evaluate the photothermal performance, MoCe@PANI NPs dispersions at various concentrations (0–400 μg/mL) were irradiated with a 1064 nm continuous-wave laser (LR-MFJ-1064/5000 mW). The temperature elevation and thermal images were recorded in real time using a Fluke TiS40 infrared thermal camera. For the photothermal stability test, four successive laser on/off cycles were performed on a single sample (400 μg/mL, 1.0 W/cm^2^, 10 min per cycle). The photothermal conversion efficiency (*η*) was determined according to Roper's method from the transient heating-cooling profile of an aqueous dispersion (400 μg/mL) under 1064 nm laser irradiation (1.0 W/cm^2^).

### 2.5 GSH depletion assay and peroxidase-like activity

To evaluate the dual redox activity, GSH depletion and peroxidase-like catalytic performance were assessed using the DTNB colorimetric assay and TMB chromogenic oxidation, respectively. For GSH depletion, MoCe@PANI NPs at gradient concentrations were incubated with GSH (100 μM) for 15 h at room temperature. DTNB (100 μM) was then introduced, and the absorbance of the resulting 5-thio-2-nitrobenzoic acid (TNB) was recorded at 412 nm; time-dependent monitoring was performed at 30-min intervals over 150 min. For peroxidase-like activity, TMB (200 μM) and H_2_O_2_ (1.0 mM) were reacted with MoCe@PANI NPs (400 μg/mL) in PBS (pH 7.4) at 37 °C for 30 min. To examine photothermal enhancement, the reaction was concurrently irradiated with a 1064 nm laser (1.0 W/cm^2^, 5 min). The absorbance of oxidized TMB at 652 nm was recorded. Hydroxyl radical generation was further verified by ESR spectroscopy using DMPO as the spin trap.

### 2.6 Steady-state kinetic analysis

Steady-state kinetics were performed at room temperature (∼25 °C), 37 °C, and 50 °C to evaluate the temperature-dependent catalytic performance. Fixed concentrations of MoCe@PANI NPs (400 μg/mL) and TMB (200 μM) were reacted with varying concentrations of H_2_O_2_ (2–120 mM) in PBS (pH 7.4). The initial reaction rate (*v*) was determined from the absorbance-time curve at 652 nm. Kinetic parameters (Michaelis constant, *K*_m_, and maximum reaction velocity, *V*_max_) were derived using the Michaelis-Menten equation and the Lineweaver-Burk double reciprocal plot.

### 2.7 Cell culture and *in vitro* cytotoxicity assay

Mouse fibroblast L929 cells and mouse breast carcinoma 4T1 cells were cultured in DMEM supplemented with 10% FBS and 1% penicillin-streptomycin at 37 °C under a 5% CO_2_ humidified atmosphere.

For cytotoxicity assessment, cells were seeded into 96-well plates (1 × 10^4^ cells/well) and cultured overnight. After incubation with MoCe@PANI NPs at a series of concentrations (0–400 μg/mL) for 12 h, cell viability was determined using the CCK-8 assay. For the PTT-CDT combination group, cells were irradiated with a 1064 nm laser (1.0 W/cm^2^, 5 min) after 4 h of co-incubation and cultured for an additional 12 h before measurement. Absorbance at 450 nm was recorded using a microplate reader. Live/dead cell staining was performed using Calcein-AM/PI double staining according to the manufacturer's protocol and imaged under a fluorescence microscope.

### 2.8 Intracellular ROS, mitochondrial function, and lipid peroxidation evaluation

4T1 cells were subjected to four treatment groups: PBS (control), PBS + Laser, MoCe@PANI NPs (400 μg/mL), and MoCe@PANI NPs + 1064 nm Laser (1.0 W/cm^2^, 5 min). For each group, total intracellular ROS was detected with DCFH-DA; mitochondrial superoxide with MitoSOX Red; mitochondrial membrane potential with JC-1 (evaluating the shift from red aggregates to green monomers); and lipid peroxidation with BODIPY 581/591 C11 (monitoring the oxidation-induced green fluorescence shift). All fluorescence images were captured using an inverted fluorescence microscope or confocal laser scanning microscope.

### 2.9 Western blot analysis

Total protein was extracted from treated 4T1 cells using RIPA lysis buffer containing protease inhibitors, and concentrations were determined by BCA assay. Equal amounts of protein (20–30 μg) were resolved by 12% SDS-PAGE and transferred onto a 0.22 μm PVDF membrane. After blocking with 5% non-fat milk in TBST, the membrane was probed with primary antibodies against GPX4 (1:1000) and GAPDH (1:2000) overnight at 4 °C, followed by HRP-conjugated secondary antibody incubation. Bands were visualized using an ECL substrate and imaged with a GelDoc Go system (Bio-Rad). Quantification was performed using ImageJ software with GAPDH as the loading control.

### 2.10 Ferroptosis inhibition rescue experiment

To determine whether cell death was ferroptosis-dependent, a rescue experiment was performed using the specific ferroptosis inhibitor Liproxstatin-1. 4T1 cells were pretreated with Liproxstatin-1 at varying concentrations (0, 2, 4, and 8 μM) for 2 h before the addition of MoCe@PANI NPs (400 μg/mL) and subsequent 1064 nm laser irradiation. After 12 h of incubation, cell viability was quantified by the CCK-8 assay.

### 2.11 Intracellular GSH and MDA quantification

Intracellular GSH levels were measured using a GSH Assay Kit. Briefly, after various treatments, cells were lysed and deproteinized. The supernatant was incubated with the chromogenic substrate in the assay buffer, and the formation rate of TNB was monitored at 412 nm. A standard curve of pure GSH was used for quantification, and GSH levels were normalized to total protein content. For the concentration-dependent GSH depletion experiment, 4T1 cells were incubated with a gradient concentration of MoCe@PANI NPs (0–400 μg/mL) for 4 h before measurement.

Malondialdehyde (MDA), a terminal product of lipid peroxidation, was quantified using an MDA Assay Kit. Cell lysates were heated with thiobarbituric acid (TBA) at 100 °C for 15 min, and the absorbance of the resulting MDA–TBA adduct was measured at 532 nm. MDA content was normalized to total protein concentration.

### 2.12 In vitro assessment of immunogenic cell death (ICD)

Immunofluorescence (IF) staining was performed to evaluate the surface exposure of calreticulin (CRT) and the release of high mobility group box 1 (HMGB1). After treatments, cells were fixed with 4% paraformaldehyde, permeabilized with 0.1% Triton X-100, and blocked with 5% BSA. Cells were incubated with anti-CRT or anti-HMGB1 primary antibodies overnight at 4 °C, followed by Alexa Fluor-conjugated secondary antibodies. Nuclei were counterstained with DAPI, and images were captured using confocal microscopy. Mean fluorescence intensity (MFI) was quantified using ImageJ software. The concentrations of secretory cytokines (IL-6 and TNF-α) and HMGB1 in the cell culture supernatant were measured using corresponding enzyme-linked immunosorbent assay (ELISA) kits according to the manufacturers’ instructions.

### 2.13 Animal models

For the unilateral tumor model, 4T1 cells (2 × 10^6^ in 100 μL PBS) were subcutaneously injected into the right flank of mice. For the bilateral tumor model, the same number of cells was injected into the right flank (primary tumor) and the left flank (distant tumor, inoculated 3 days later). When tumor volumes reached approximately 100 mm^3^, mice were randomly divided into different treatment groups.

### 2.14 In vivo photoacoustic imaging and biodistribution

To track the *in vivo* distribution of MoCe@PANI NPs, photoacoustic (PA) imaging was performed using a MSOT inVision 128 system. For *in vitro* phantom imaging, different concentrations of MoCe@PANI NPs (0–400 μg/mL) were embedded in agar phantoms. For *in vivo* imaging, tumor-bearing mice were anesthetized and scanned before and at predetermined time points (0, 4, 12, 24, 48, and 72 h) post-intravenous injection of MoCe@PANI NPs (10 mg/kg). The PA signal intensity of the tumor region was quantitatively analyzed.

### 2.15 In vivo photothermal therapy and antitumor efficacy

Mice bearing 4T1 tumors were randomly divided into four groups (n = 4 per group for the unilateral model): (1) PBS, (2) PBS + Laser, (3) MoCe@PANI NPs, and (4) MoCe@PANI NPs + Laser. For the unilateral model, mice received an intravenous injection of MoCe@PANI NPs (10 mg/kg, 200 μL) or an equivalent volume of PBS. Twenty-four hours post-injection, the tumor site was irradiated with a 1064 nm laser (2.0 W/cm^2^, 10 min). For the bilateral model, MoCe@PANI NPs were injected intratumorally into the primary (right-sided) tumor, and laser irradiation was performed 24 h post-injection. The contralateral (left-sided) tumor was left untreated as a negative control to evaluate the abscopal effect.

Tumor volumes were calculated as V = (length × width^2^)/2 and measured every two days. Body weight was monitored as an indicator of systemic toxicity. On day 14, mice were euthanized, and tumors were excised, weighed, and photographed.

### 2.16 Flow cytometry analysis of the tumor immune microenvironment

To analyze the immunomodulatory effects, tumors were harvested on day 3 post-treatment. Single-cell suspensions were prepared by mechanically dissociating tumor tissues and digesting them with collagenase I (1 mg/mL) and DNase I (200 μg/mL) for 30–45 min at 37 °C. After filtration through a 70 μm cell strainer and erythrocyte lysis, the cells were blocked with anti-CD16/32 antibody and stained with the corresponding fluorochrome-conjugated antibodies for 30 min at 4 °C. Fluorescence data were acquired on a CytoFLEX flow cytometer and analyzed using FlowJo software. The gating strategy identified: mature DCs (CD80^+^CD86^+^), CD8^+^ and CD4^+^ T cells, activated CD8^+^ T cells (CD8^+^CD69^+^), M1 macrophages (F4/80^+^CD80^+^), M2 macrophages (CD163^+^CD206^+^), and myeloid-derived suppressor cells (MDSCs, CD11b^+^Gr-1^+^).

### 2.17 Histological and immunohistochemical analysis

Harvested tumors and major organs (heart, liver, spleen, lung, kidney) were fixed in 4% paraformaldehyde, embedded in paraffin, and sectioned at 4 μm thickness. Hematoxylin and eosin (H&E) staining was performed to evaluate histological morphology. For proliferative activity assessment, immunohistochemical staining against Ki-67 was carried out. The sections were incubated with anti-Ki-67 primary antibody overnight at 4 °C, followed by HRP-conjugated secondary antibody and DAB chromogenic detection. Hematoxylin was used for nuclear counterstaining.

### 2.18 Biosafety evaluation

To assess potential systemic toxicity, blood samples were collected from mice at the experimental endpoint for hematological analysis (white blood cells, WBC; platelets, PLT; hemoglobin, HGB) and serum biochemical assays (alanine aminotransferase, ALT; aspartate aminotransferase, AST; blood urea nitrogen, BUN). Hemolytic activity was also tested by incubating the nanoparticles with a diluted erythrocyte suspension, and the absorbance of the supernatant was measured at 540 nm.

### 2.19 Statistical analysis

All experiments were carried out independently at least three times, and data are presented as mean ± standard deviation (SD). Statistical comparisons between two groups were performed using a two-tailed unpaired Student's t-test, while comparisons among multiple groups were determined by one-way analysis of variance (ANOVA) followed by Tukey's post-hoc test. All statistical analyses and graph generation were performed using GraphPad Prism 10 and OriginPro 2026 software. P-values <0.05 were considered statistically significant (*p < 0.05, **p < 0.01, ***p < 0.001).

## 3 Results and discussion

### 3.1 Preparation and characterization of MoCe@PANI NPs

MoCe@PANI NPs with different metal precursor amounts (25, 100, and 200 mg), as well as control materials free PANI (without Mo/Ce) and free MoCe (without PANI), were synthesized via a one-pot oxidative polymerization method.

**Fig. 1:**
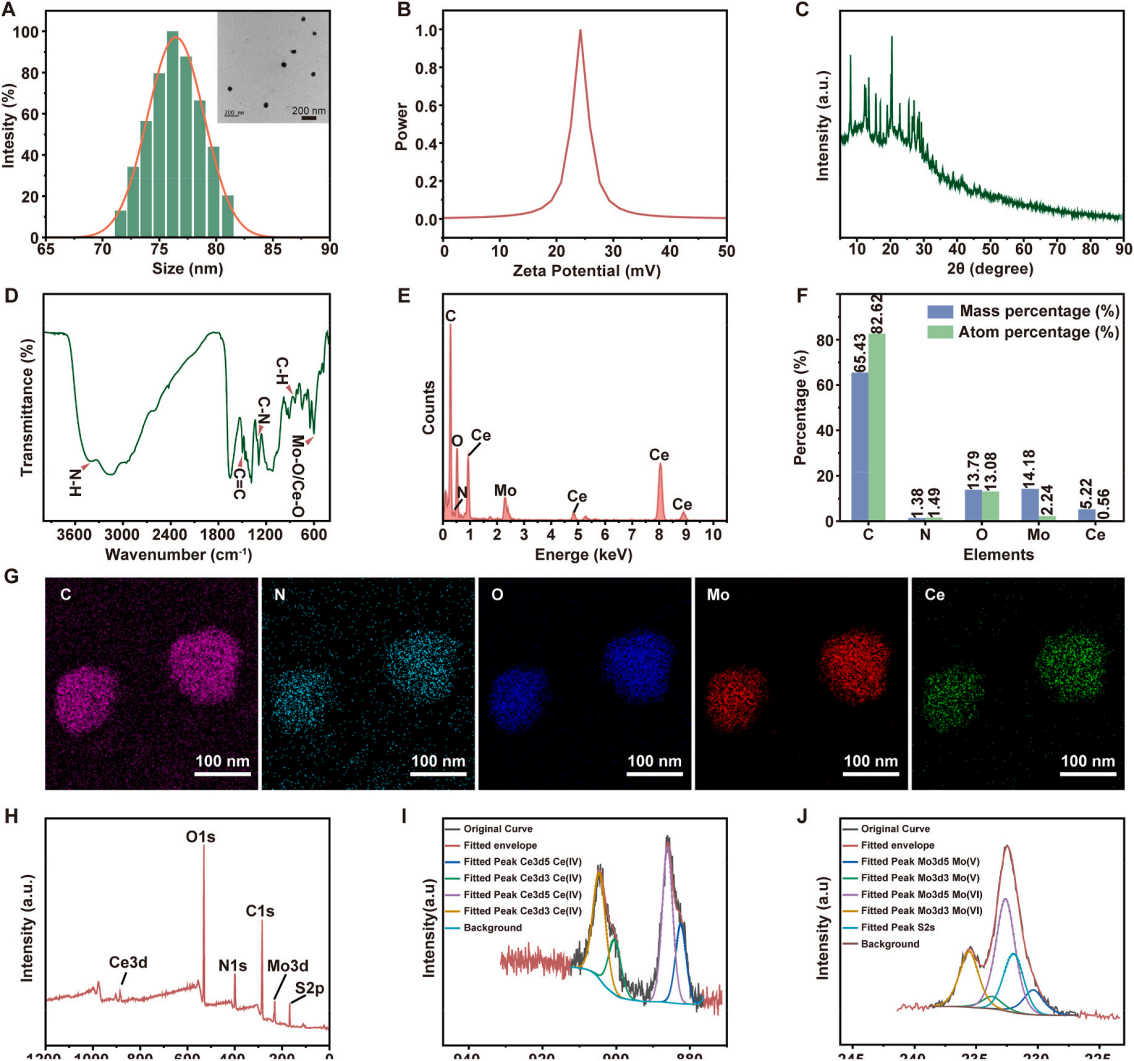
Physicochemical characterization of MoCe@PANI NPs. (A) TEM image (inset) and DLS size distribution. Scale bar: 200 nm. (B) *Zeta* potential. (C) XRD pattern. (D) FTIR spectrum. (E) EDS spectrum. (F) Mass and atomic percentages of constituent elements from EDS analysis. (G) Elemental mapping images. Scale bars: 100 nm. (H) XPS survey spectrum. (I) High-resolution XPS spectrum of Ce3d. (J) High-resolution XPS spectrum of Mo3d.

The morphology and size of the as-synthesized MoCe@PANI NPs were characterized by transmission electron microscopy (TEM) and dynamic light scattering (DLS). As shown in Fig. 1A, the TEM image (inset) revealed a uniform spherical morphology, while the DLS measurement indicated a narrow size distribution with an average hydrodynamic diameter of approximately 76 nm. Comparison of the different metal loadings (Fig. S1) revealed that the 100 mg formulation exhibited the most uniform spherical morphology with minimal aggregation, whereas both the 25 mg and 200 mg formulations showed varying degrees of particle aggregation and irregular shapes, confirming the optimal colloidal properties of the original composition. The *zeta* potential of MoCe@PANI NPs was measured to be +22.4 mV (Fig. 1B), which is attributed to the protonation of imine groups on the PANI backbone under acidic synthetic conditions. To evaluate the colloidal stability, the hydrodynamic size was monitored over 14 days at room temperature, with no significant change observed (Fig. S2). Furthermore, TEM imaging of nanoparticles after incubation in PBS (pH 7.4) for 7 days showed no appreciable aggregation or morphological changes (Fig. S3), confirming their structural integrity under physiological conditions.

The crystalline structure and chemical composition were investigated by X-ray diffraction (XRD) and Fourier transform infrared (FTIR) spectroscopy. The XRD pattern (Fig. 1C) displayed characteristic diffraction peaks corresponding to the semi-crystalline structure of PANI along with specific crystal planes of cerium molybdate, confirming the successful incorporation of the inorganic dopants. In the FTIR spectrum (Fig. 1D), a sharp band at 1497 cm^−1^ was assigned to the C=C stretching vibration of the benzenoid rings of PANI. Notably, the characteristic absorption band of the quinoid ring (typically at ∼1575 cm^−1^) was absent, indicating that the PANI backbone exists predominantly in the reduced state. Nevertheless, an intense band at 1138 cm^−1^, attributed to polaron lattice vibration, confirmed efficient doping and good electrical conductivity of the polymer matrix. The band at 1302 cm^−1^ (C–N stretching) along with a shoulder at 1236 cm^−1^ (C–N^+^·) further substantiated the doped state. A band at 820 cm^−1^ was assigned to the out-of-plane bending vibration of para-substituted benzene rings, confirming the linear structure of PANI. The broad band at 594 cm^−1^ was assigned to Mo–O/Ce–O vibrations, verifying the successful incorporation of cerium molybdate. The significantly intensified N–H stretching band at 3414 cm^−1^ suggested strong coordination between metal ions (Ce^4+^, Mo^6+^) and the PANI chains. No impurity bands (e.g., carbonyl at ∼1700 cm^−1^) were detected.

Energy-dispersive X-ray spectroscopy (EDS) and elemental mapping were employed to analyze the elemental composition. The EDS spectrum (Fig. 1E) clearly identified the presence of C, N, O, Mo, and Ce elements, confirming the successful introduction of all components. Quantitative analysis (Fig. 1F) showed that carbon (C) accounted for the highest proportion (65.43 wt%, 82.62 at%), consistent with the dominant PANI organic framework. The presence of nitrogen (N, 13.08 wt%) verified the integrity of the polymer structure. Molybdenum (Mo) and cerium (Ce) were detected at 14.18 wt%/2.24 at% and 5.22 wt%/0.56 at%, respectively, confirming the effective incorporation of both metal elements. Elemental mapping images (Fig. 1G) demonstrated a homogeneous spatial distribution of C, N, O, Mo, and Ce throughout the nanoparticles, with no obvious aggregation or segregation, indicating molecular-level uniform doping rather than physical adsorption. Inductively coupled plasma mass spectrometry (ICP-MS) was further employed to precisely quantify the metal loading. The concentrations of Mo and Ce in the as-prepared dispersion were determined to be 92.0 ± 1.0 μg/kg and 122.0 ± 1.0 μg/kg, respectively, corresponding to mass percentages of 9.20 × 10^−6^% and 1.22 × 10^−5^% (based on the original aqueous dispersion; Table S1). When normalized to the mass of lyophilized nanoparticles, the actual loading amounts of Mo and Ce were calculated to be 0.092 μg/mg and 0.122 μg/mg, respectively.

X-ray photoelectron spectroscopy (XPS) was performed to investigate the surface chemical states and elemental composition. The survey spectrum (Fig. 1H) confirmed the presence of C1s, N1s, O1s, Mo3d, and Ce3d signals, consistent with the EDS results. A weak S2s signal was also detected, attributed to residual sulfate species from the APS oxidant, indicating their successful incorporation as dopants on the polymer surface. High-resolution XPS analysis of the Ce3d region (Fig. 1I) revealed complex spin-orbit splitting multiplets, characteristic of cerium-containing species. The peaks located at ∼882.51, 886.11, 900.43, and 904.57 eV were all attributed to Ce^4+^ species, whereas no distinct signals corresponding to Ce^3+^ (typically at ∼880.5 and ∼899.0 eV) were observed. This indicated that cerium predominantly exists in the +4 oxidation state within the composite, endowing the material with strong oxidizing capability for GSH depletion via redox reactions. In contrast, the Mo 3d spectrum (Fig. 1J) revealed the coexistence of mixed valence states. The doublet peaks at 232.62 and 235.57 eV were assigned to Mo^6+^, while additional peaks at 230.37 and 233.72 eV corresponded to Mo^5+^. The presence of Mo^5+^/Mo^6+^ couples suggested the formation of oxygen vacancies and electron transfer channels, which could serve as active sites for Fenton-like catalytic reactions. Collectively, these characterizations confirmed the successful fabrication of the MoCe@PANI nanoplatform with a well-defined composition and an optimized electronic structure.

The optical absorption properties of the different formulations were further compared by UV-vis-NIR spectroscopy (Fig. S4). Free PANI exhibited strong NIR-II absorption comparable to MoCe@PANI, while free MoCe showed negligible absorbance at 1064 nm, confirming that PANI serves as the photothermal component. Among the three metal loadings, the 100 mg formulation displayed the highest absorbance at 1064 nm, supporting its superior photothermal performance.

### 3.2 NIR-II photothermal performance of MoCe@PANI NPs

**Fig. 2:**
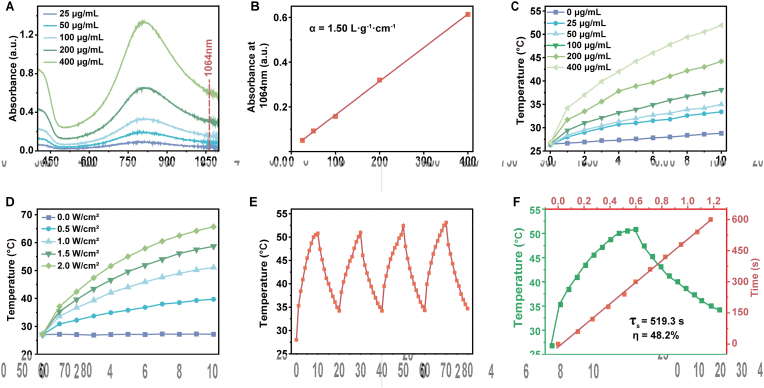
NIR-II photothermal performance of MoCe@PANI NPs. (A) UV-vis-NIR absorption spectra at various concentrations. (B) Linear fitting of absorbance at 1064 nm versus concentration for determining the mass extinction coefficient. (C) Concentration-dependent temperature elevation curves under 1064 nm laser irradiation (1.0 W/cm^2^). (D) Power density-dependent temperature elevation curves of MoCe@PANI NPs (400 μg/mL). (E) Temperature variation curves over four consecutive laser on/off cycles. (F) Time-dependent temperature change during a single heating-cooling cycle under 1064 nm laser irradiation (green curve) and linear fitting of the cooling period (red line) for calculating the photothermal conversion efficiency. Data are presented as mean ± SD (n = 3).

The optical absorption properties of MoCe@PANI NPs were evaluated by UV-vis-NIR spectroscopy. As shown in Fig. 2A, the nanoparticles exhibited broad and concentration-dependent absorption across the visible to NIR-II region (700–1100 nm), attributable to the electronic transitions within the conjugated PANI backbone enhanced by metal ion doping. A linear correlation between the absorbance at 1064 nm and nanoparticle concentration was established (Fig. 2B), yielding a high mass extinction coefficient (*α*) of 1.50 L g^−1^ cm^−1^.

The photothermal conversion performance was subsequently investigated under 1064 nm laser irradiation. In the concentration-dependent study (1.0 W/cm^2^), the temperature elevation exhibited a clear dose-dependent behavior (Fig. 2C). At a concentration of 400 μg/mL, the dispersion temperature rapidly increased from ambient temperature to over 50 °C within 10 min. Corresponding infrared thermal images (Fig. S5) visually confirmed the concentration-dependent spatial heat generation. The power density dependence was further examined at a fixed concentration of 400 μg/mL. As the laser power density increased from 0.5 to 2.0 W/cm^2^, the temperature elevation rate and maximum temperature increased accordingly (Fig. 2D and S6), with the temperature reaching approximately 55 °C within 5 min at 2.0 W/cm^2^.

Photothermal stability was assessed by four consecutive laser on/off cycles. The temperature variation curves (Fig. 2E) demonstrated highly reproducible heating-cooling profiles with negligible attenuation in the maximum temperature, indicating excellent photothermal reversibility and long-term thermal stability. The photothermal conversion efficiency (*η*) was calculated based on the energy balance model using the cooling curve data (Fig. 2F). The time constant (*τ*_s_) was derived from the linear fitting of the cooling period, and *η* was determined to be 48.2%, which is superior to many previously reported organic photothermal agents.

### 3.3 GSH depletion and Fenton-like catalytic activity of MoCe@PANI NPs

**Fig. 3:**
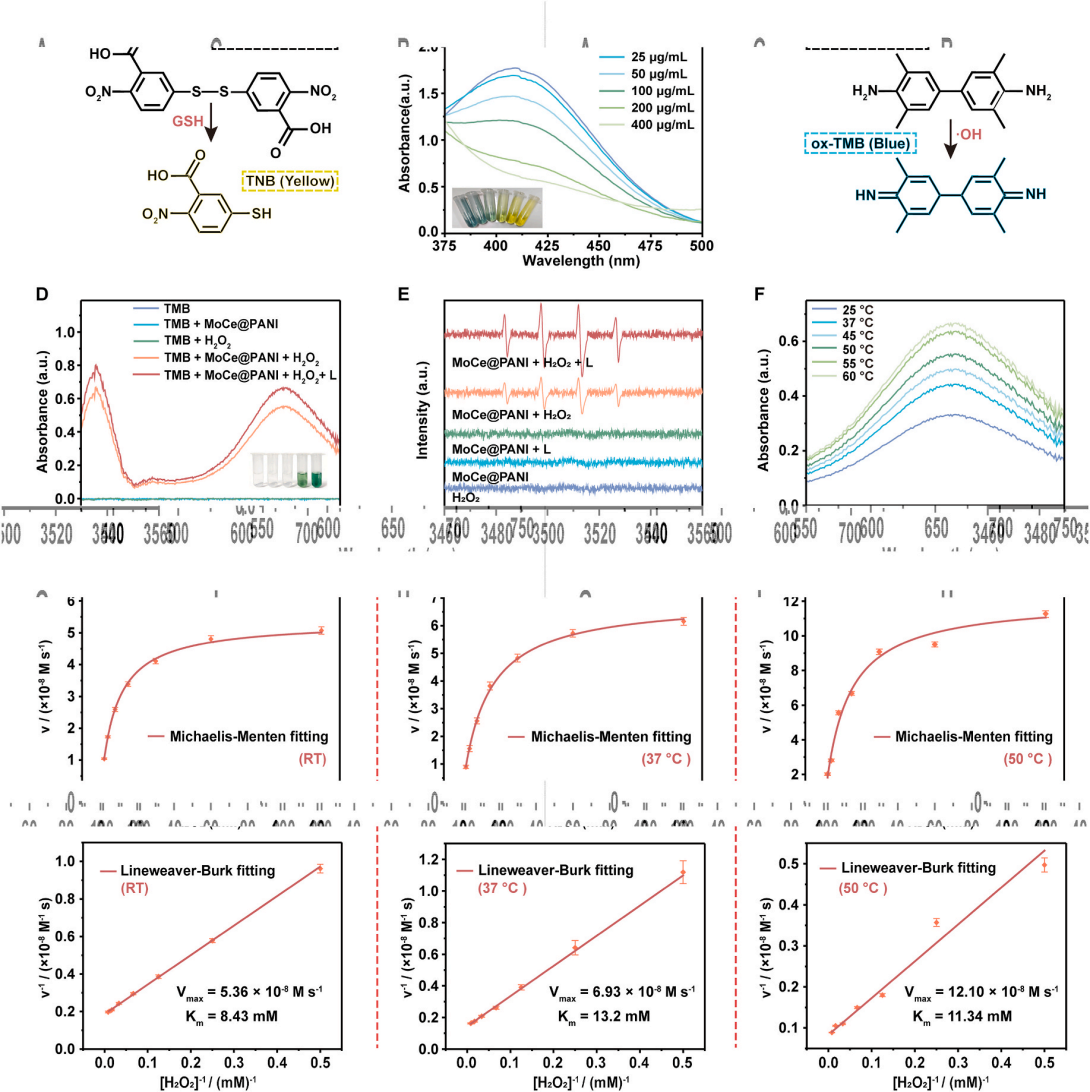
Redox activity and Fenton-like catalytic performance of MoCe@PANI NPs. (A) Schematic illustration of the colorimetric reaction between GSH and DTNB. (B) UV-vis absorption spectra showing GSH depletion by MoCe@PANI NPs at various concentrations. (C) Schematic illustration of the chromogenic oxidation of TMB catalyzed by ·OH. (D) UV-vis absorption spectra of TMB oxidation under different conditions. (E) ESR spectra of ·OH generation. (F) Temperature-dependent UV-vis absorption spectra of oxidized TMB (25-60 °C). (G–I) Steady-state kinetic analysis at (G) room temperature, (H) 37 °C, and (I) 50 °C, with Michaelis-Menten fitting curves (top) and Lineweaver-Burk plots (bottom). Data are presented as mean ± SD (n = 3).

The therapeutic efficacy of chemodynamic therapy (CDT) is often compromised by elevated intracellular levels of GSH, which act as a potent ROS scavenger. Therefore, the GSH-depleting capability of MoCe@PANI NPs was first evaluated using the DTNB colorimetric assay. As illustrated in Fig. 3A, GSH reacts with DTNB to generate the yellow-colored product 2-nitro-5-thiobenzoate (TNB), providing a reliable basis for quantitative GSH detection. Upon incubation with increasing concentrations of MoCe@PANI NPs (0–400 μg/mL), the characteristic absorption peak of TNB at 412 nm decreased progressively (Fig. 3B), confirming efficient and concentration-dependent depletion of GSH. Time-dependent measurements further demonstrated that the absorbance at 412 nm continued to decline over 180 min, with the most rapid decrease occurring within the first 60 min (Fig. S7), indicating sustained GSH-depleting activity of the nanoparticles.

The Fenton-like catalytic activity of MoCe@PANI NPs in generating hydroxyl radicals (·OH) was subsequently assessed using TMB as a chromogenic substrate. As illustrated in Fig. 3C, colorless TMB is oxidized by ·OH to blue ox-TMB, which exhibits characteristic absorption peaks at 370 nm and 652 nm. As shown in Fig. 3D, significant absorption peaks were observed exclusively in the presence of both MoCe@PANI NPs and H_2_O_2_, whereas control groups of TMB alone, TMB + MoCe@PANI, or TMB + H_2_O_2_ exhibited negligible signals. Notably, the introduction of 1064 nm laser irradiation (”+ L”) to the complete reaction system further enhanced the absorbance intensity, indicating that photothermal heating promotes the Fenton-like catalytic efficiency. The corresponding color change of the TMB solution (inset) provided direct visual confirmation of ·OH generation.

To further verify the generation of ·OH, electron spin resonance (ESR) spectroscopy was performed using DMPO as the spin trap. As shown in Fig. 3E, a characteristic 1:2:2:1 quartet signal corresponding to DMPO-·OH adducts was detected in the MoCe@PANI + H_2_O_2_ group, while no discernible signals were observed for MoCe@PANI alone or MoCe@PANI + Laser, confirming that ·OH production is specifically catalyzed by MoCe@PANI NPs in the presence of H_2_O_2_.

Given that photothermal therapy can elevate local temperatures, the effect of hyperthermia on the catalytic performance of MoCe@PANI NPs was investigated. As shown in Fig. 3F, the absorbance intensity of oxidized TMB increased steadily as the temperature was elevated from 25 to 60 °C, indicating that heat significantly accelerates the Fenton-like reaction kinetics. To quantitatively assess this thermal enhancement, steady-state kinetic analyses were performed at room temperature (RT), 37 °C, and 50 °C. The Michaelis-Menten fitting curves (Fig. 3G–I, top panels) and corresponding Lineweaver-Burk plots (Fig. 3G–I, bottom panels) revealed that the maximum reaction velocity (*V*_max_) increased substantially with temperature (from 3.96 × 10^−8^ s^−1^ at RT to 12.10 × 10^−8^ M s^−1^ at 50 °C), representing an approximately 3.05-fold enhancement. The Michaelis constant (*K*_m_) remained relatively stable across the temperature range, indicating that the elevated temperature primarily enhanced the catalytic turnover rate rather than the substrate-binding affinity.

### 3.4 In vitro antitumor efficacy and ferroptosis mechanism of MoCe@PANI NPs

**Fig. 4:**
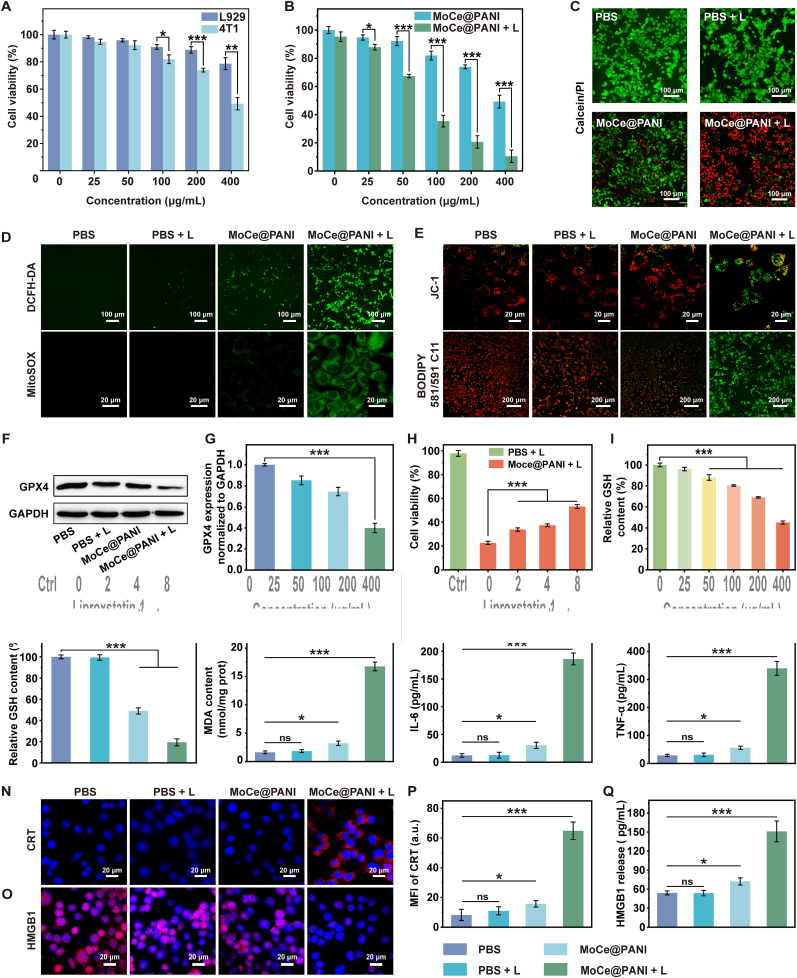
*In vitro* antitumor efficacy and ferroptosis mechanism of MoCe@PANI NPs. (A) Cell viability of L929 and 4T1 cells after incubation with MoCe@PANI NPs at various concentrations. (B) Cell viability of 4T1 cells treated with MoCe@PANI NPs with or without 1064 nm laser irradiation (1.0 W/cm^2^, 5 min). (C) Live/dead staining of 4T1 cells (Calcein-AM/PI) after different treatments. (D) DCFH-DA staining (total ROS) and MitoSOX staining (mitochondrial superoxide). (E) JC-1 staining (mitochondrial membrane potential) and BODIPY 581/591 C11 staining (lipid peroxidation). (F) Western blot analysis of GPX4 protein expression. (G) Quantification of GPX4 band intensity relative to GAPDH. (H) Cell viability of 4T1 cells treated with MoCe@PANI + Laser in the presence of increasing concentrations of Liproxstatin-1. (I) Intracellular GSH levels after incubation with increasing concentrations of MoCe@PANI NPs. (J) Relative GSH content and (K) MDA content in 4T1 cells after different treatments. (L) IL-6 and (M) TNF-α levels in cell culture supernatants. (N) Immunofluorescence staining of CRT (red) and (O) HMGB1 (red). Nuclei stained with DAPI (blue). (P) Mean fluorescence intensity (MFI) of CRT surface exposure. (Q) HMGB1 release levels. Data are presented as mean ± SD (n = 3). *p < 0.05, **p < 0.01, ***p < 0.001; ns, not significant.

Before evaluating the antitumor efficacy, the biocompatibility of MoCe@PANI NPs was first assessed using normal fibroblast L929 cells. As shown in Fig. 4A, cell viability remained above 80% even at a concentration of 400 μg/mL after 12 h incubation, indicating favorable biosafety. In contrast, MoCe@PANI NPs exhibited significant concentration-dependent cytotoxicity toward 4T1 breast cancer cells, which was further amplified under 1064 nm laser irradiation (1.0 W/cm^2^, 5 min) (Fig. 4B). At 400 μg/mL with laser treatment, cell viability decreased to approximately 10%, demonstrating a potent synergistic therapeutic effect. These results were visually confirmed by Calcein-AM/PI live/dead staining (Fig. 4C).

To further validate the synergistic mechanism and optimize the component ratio, we performed control experiments with free PANI, free MoCe, and MoCe@PANI with different metal loadings (25, 100, and 200 mg). As shown in Fig. S8, the combination of MoCe@PANI (100 mg) with laser achieved ∼90% cell killing, which is significantly higher than the additive effect of free PANI + Laser and free MoCe + Laser, confirming a genuine synergistic interaction. Among the three metal loadings, the 100 mg formulation exhibited the strongest anticancer activity. TMB assays further confirmed that the 100 mg formulation produced the highest ·OH level under laser irradiation (Fig. S9). These data demonstrate that the 100 mg composition offers the optimal balance of catalytic activity and synergistic efficacy.

To elucidate the underlying mechanism, intracellular ROS generation and mitochondrial function were investigated. Fluorescence imaging (Fig. 4D) revealed that the MoCe@PANI + Laser group exhibited the strongest fluorescence signals for DCFH-DA (total ROS) and MitoSOX (mitochondrial superoxide), confirming that the combination therapy maximizes oxidative stress. Concurrently, JC-1 staining (Fig. 4E, upper panel**)** showed a distinct shift from red aggregates to green monomers, indicating severe mitochondrial membrane potential (MMP) collapse. Furthermore, BODIPY 581/591 C11 staining (Fig. 4E, lower panel**)** displayed intense green fluorescence, suggesting extensive lipid peroxidation, a hallmark of ferroptosis.

To verify the ferroptotic mechanism, Western blot analysis was performed to detect glutathione peroxidase 4 (GPX4), a key negative regulator of ferroptosis. As shown in Fig. 4F and G, GPX4 expression was markedly downregulated in the combined treatment group. Additionally, a rescue experiment using Liproxstatin-1, a specific ferroptosis inhibitor, partially restored cell viability in a dose-dependent manner (Fig. 4H), confirming that ferroptosis is the primary mode of cell death.

The depletion of intracellular GSH was systematically quantified. A concentration-dependent decrease in GSH levels was observed upon incubation with MoCe@PANI NPs, dropping to approximately 45% at 400 μg/mL (Fig. 4I). Across the four treatment groups (Fig. 4J), laser irradiation alone had negligible effect, while MoCe@PANI NPs alone significantly reduced GSH content. The combination of NPs and laser irradiation resulted in the most substantial GSH depletion (∼20% remaining), which was attributed to the photothermal acceleration of redox reactions. This profound GSH depletion contributed to elevated lipid peroxidation, as evidenced by the significantly higher malondialdehyde (MDA) levels in the combined treatment group compared to controls (Fig. 4K).

The potential of MoCe@PANI NPs to induce immunogenic cell death (ICD) was further evaluated. ELISA results showed that the combined treatment significantly upregulated the secretion of pro-inflammatory cytokines IL-6 (Fig. 4L) and TNF-α (Fig. 4M) in cell culture supernatants. Immunofluorescence staining (Fig. 4N) revealed markedly enhanced CRT exposure (red fluorescence) on the cell surface in the MoCe@PANI + Laser group, while HMGB1 (also red fluorescence, Fig. 4O) was predominantly confined to the nucleus in untreated control cells but largely diminished in the combined treatment group, indicating its release from the nucleus into the extracellular space. Quantitative analysis confirmed a significant increase in CRT surface exposure (Fig. 4P) and a corresponding decrease in intracellular HMGB1 retention (Fig. 4Q) in the MoCe@PANI + Laser group.

### 3.5 In vitro and in vivo PA imaging and biosafety evaluation of MoCe@PANI NPs

**Fig. 5:**
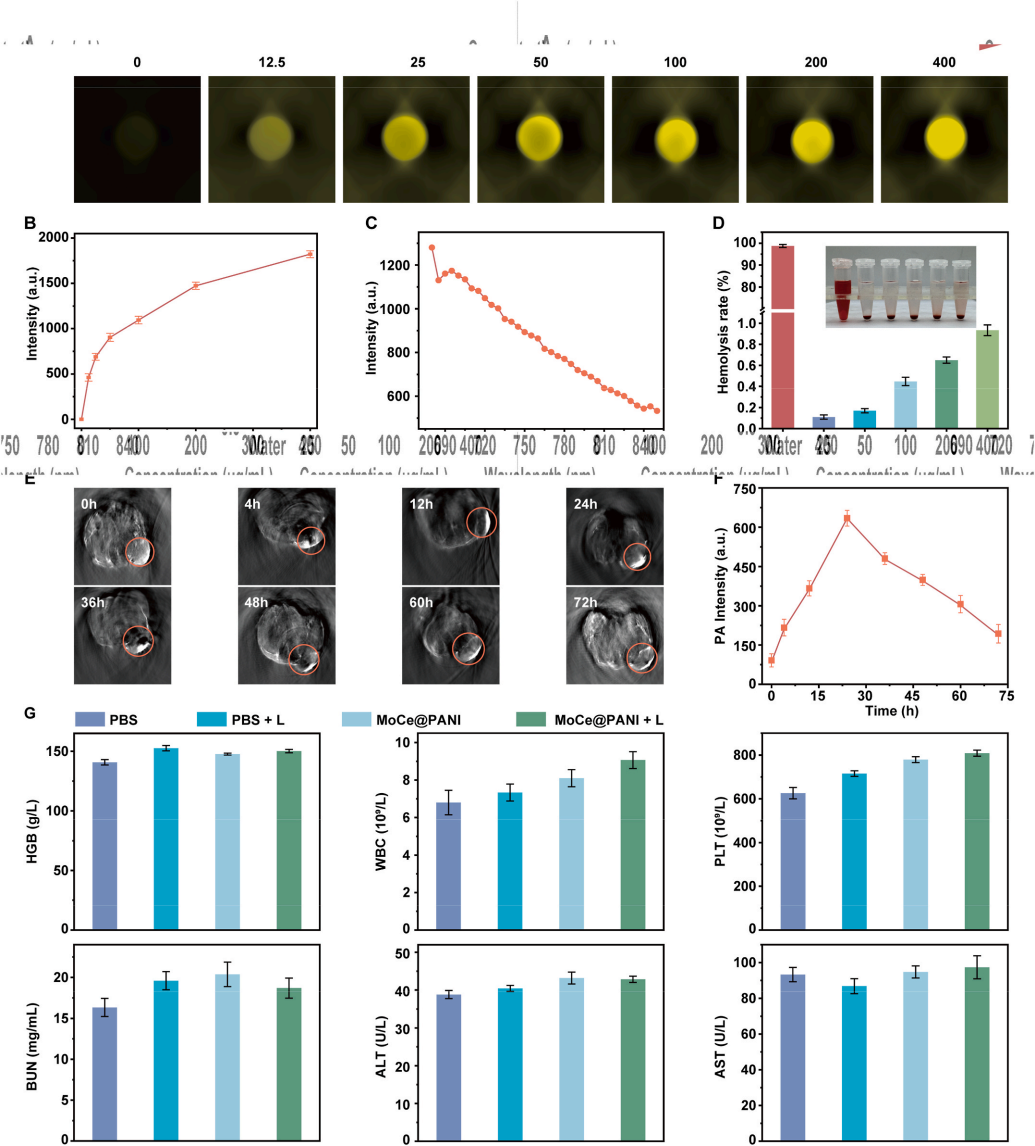
*In vitro* and *in vivo* PA imaging and biosafety evaluation of MoCe@PANI NPs. (A) Concentration-dependent PA imaging in phantom tubes. (B) Quantitative analysis of PA signal intensity versus concentration. (C) PA spectrum in the NIR-II region (680-850 nm). (D) Hemolysis assay with quantitative hemolysis ratios. (E) Time-dependent *in vivo* PA images of 4T1 tumor-bearing mice after intravenous injection of MoCe@PANI NPs. Red circles indicate the tumor region. (F) Quantitative analysis of PA signal intensity in tumors at different time points post-injection. (G) Hematological and biochemical analyses of mice after different treatments, including HGB, WBC, PLT, BUN, ALT, and AST levels. Data are presented as mean ± SD (n = 3). *p < 0.05, **p < 0.01, ***p < 0.001.

Given the strong NIR-II absorption properties of MoCe@PANI NPs, their potential as contrast agents for photoacoustic (PA) imaging was first evaluated *in vitro* using phantom tubes at concentrations ranging from 0 to 400 μg/mL. The PA signal intensity increased linearly with nanoparticle concentration (Fig. 5A and B), and the PA spectrum in the 680–850 nm region exhibited broad absorption (Fig. 5C), confirming the suitability of MoCe@PANI NPs for deep-tissue PA imaging.

Encouraged by these results, *in vivo* PA imaging was performed in 4T1 tumor-bearing mice at various time points after intravenous injection. PA signals in the tumor region gradually increased, reaching a maximum at 24 h and subsequently declining (Fig. 5E and F), indicating effective tumor accumulation via the EPR effect followed by gradual clearance.

To quantitatively validate the PA imaging findings, the biodistribution of MoCe@PANI NPs was assessed by ICP-MS measurement of Ce and Mo content in major organs and tumor at 6, 12, 24, and 48 h post-injection (Fig. S10). Both elements showed similar distribution patterns. Tumor accumulation of Ce reached 15.8% ID/g at 24 h, consistent with the PA imaging peak. Liver and spleen showed the highest uptake among normal organs (Liver: 28.5–32.1% ID/g; Spleen: 18.2–20.5% ID/g at 6–12 h), attributable to reticuloendothelial system clearance, with liver Ce content gradually declining to 18.3% ID/g by 48 h, indicating progressive hepatic elimination. Importantly, the Ce/Mo ratio remained constant across all tissues and time points (∼1.33), matching the loading ratio in the as-synthesized NPs, confirming their *in vivo* structural integrity without significant metal ion dissociation. Heart, lung, and kidney showed consistently low retention (Heart: 1.5–3.2% ID/g; Lung: 3.2–5.8% ID/g; Kidney: 5.0–8.5% ID/g), suggesting minimal nonspecific accumulation.

Finally, the biosafety of MoCe@PANI NPs was evaluated. Hemolysis remained negligible at concentrations up to 400 μg/mL (Fig. 5D), confirming excellent hemocompatibility. Hematological and serum biochemical analyses after treatment revealed that HGB, WBC, PLT, BUN, ALT, and AST levels all remained within normal physiological ranges, with no significant differences from the PBS control (Fig. 5G). Collectively, the PA imaging, ICP-MS biodistribution, and systemic toxicity data demonstrate that MoCe@PANI NPs achieve efficient tumor accumulation via the EPR effect while maintaining structural integrity and a favorable biosafety profile.

### 3.6 Synergistic chemodynamic and photothermal therapy for primary tumor ablation

**Fig. 6:**
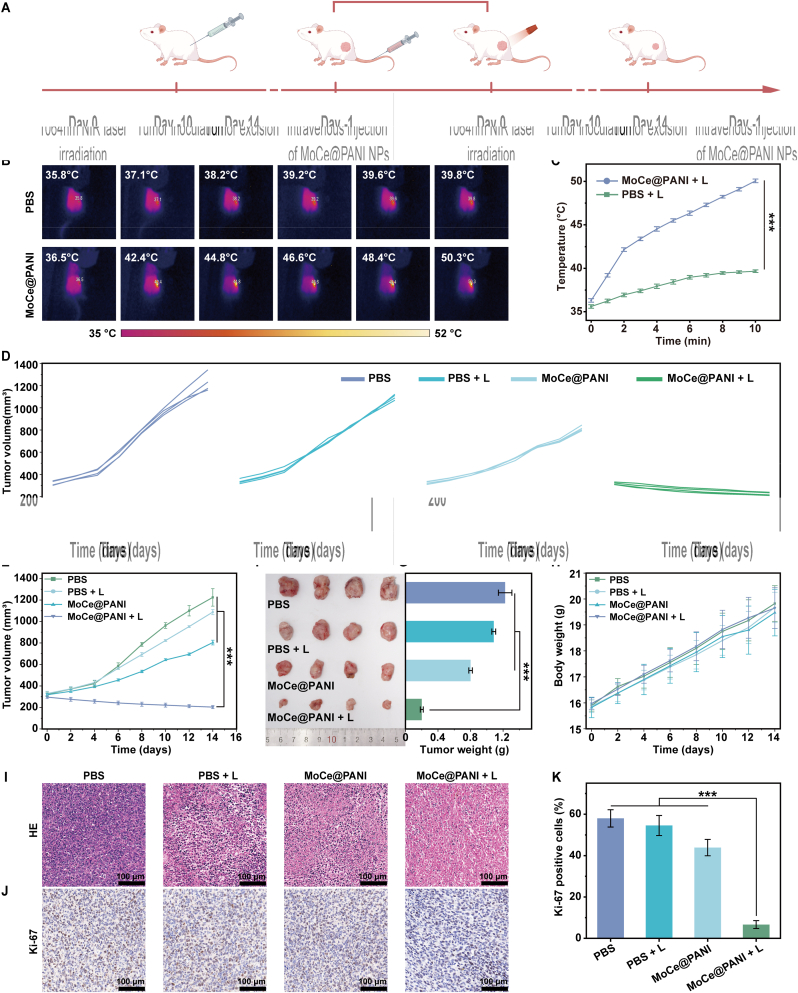
*In vivo* photothermal therapeutic efficacy of MoCe@PANI NPs in 4T1 tumor-bearing mice. (A) Schematic illustration of the experimental timeline and treatment protocol. (B) Infrared thermal images of tumors during 1064 nm laser irradiation (2.0 W/cm^2^, 10 min). (C) Corresponding temperature elevation curves at the tumor site. (D) Individual tumor volume growth curves under different treatments over 14 days. (E) Average tumor volume curves at the endpoint. (F) Representative photographs of resected tumors from each group. (G) *Ex vivo* tumor weights at the endpoint. (H) Body weight profiles of mice throughout the treatment period. (I) H&E staining of tumor sections. (J) Ki-67 immunohistochemical staining of tumor sections. (K) Quantitative analysis of Ki-67-positive cells in tumor sections. Data are presented as mean ± SD (n = 4). *p < 0.05, **p < 0.01, ***p < 0.001.

Encouraged by the excellent *in vitro* performance, the *in vivo* antitumor efficacy of MoCe@PANI NPs was evaluated in a 4T1 tumor-bearing mouse model. The experimental timeline and treatment protocol are illustrated in Fig. 6A. Mice were intravenously injected with MoCe@PANI NPs on Day −1, followed by 1064 nm NIR laser irradiation (2.0 W/cm^2^, 10 min) on the tumor site on Day 0, and tumor growth was monitored for 14 days.

To verify the *in vivo* photothermal conversion capability, infrared thermal imaging was performed during laser irradiation. As shown in Fig. 6B and C, the tumor temperature in mice injected with MoCe@PANI NPs increased rapidly, reaching 50.3 °C within 10 min, whereas the PBS control group exhibited only a modest temperature elevation to approximately 39.8 °C under the same conditions.

The therapeutic efficacy was assessed by monitoring tumor volume changes. Individual tumor growth trajectories (Fig. 6D) and average tumor volume curves (Fig. 6E) revealed that tumors in the PBS, PBS + Laser, and MoCe@PANI alone groups grew rapidly, while the MoCe@PANI + Laser group demonstrated significant tumor suppression. Representative photographs of resected tumors (Fig. 6F) and *ex vivo* tumor weights (Fig. 6G) at the endpoint further confirmed the superior antitumor efficacy of the combined treatment. Body weight monitoring throughout the treatment period showed no significant loss in any group (Fig. 6H), and mice injected with varying doses of MoCe@PANI NPs (0–40 mg/kg) maintained stable body weights (Fig. S11), indicating good tolerance.

Histological analysis was performed on tumor sections. H&E staining (Fig. 6I) revealed extensive nuclear fragmentation and coagulative necrosis in the MoCe@PANI + Laser group, whereas control groups displayed intact cellular morphology. Ki-67 immunohistochemical staining (Fig. 6J) showed markedly reduced positive signals in the combined treatment group, which was quantitatively confirmed (Fig. 6K). H&E staining of major organs at the endpoint showed no appreciable pathological abnormalities across all groups (Fig. S12).

### 3.7 Local MoCe@PANI NPs-Mediated synergistic therapy elicits systemic abscopal antitumor immunity

**Fig. 7:**
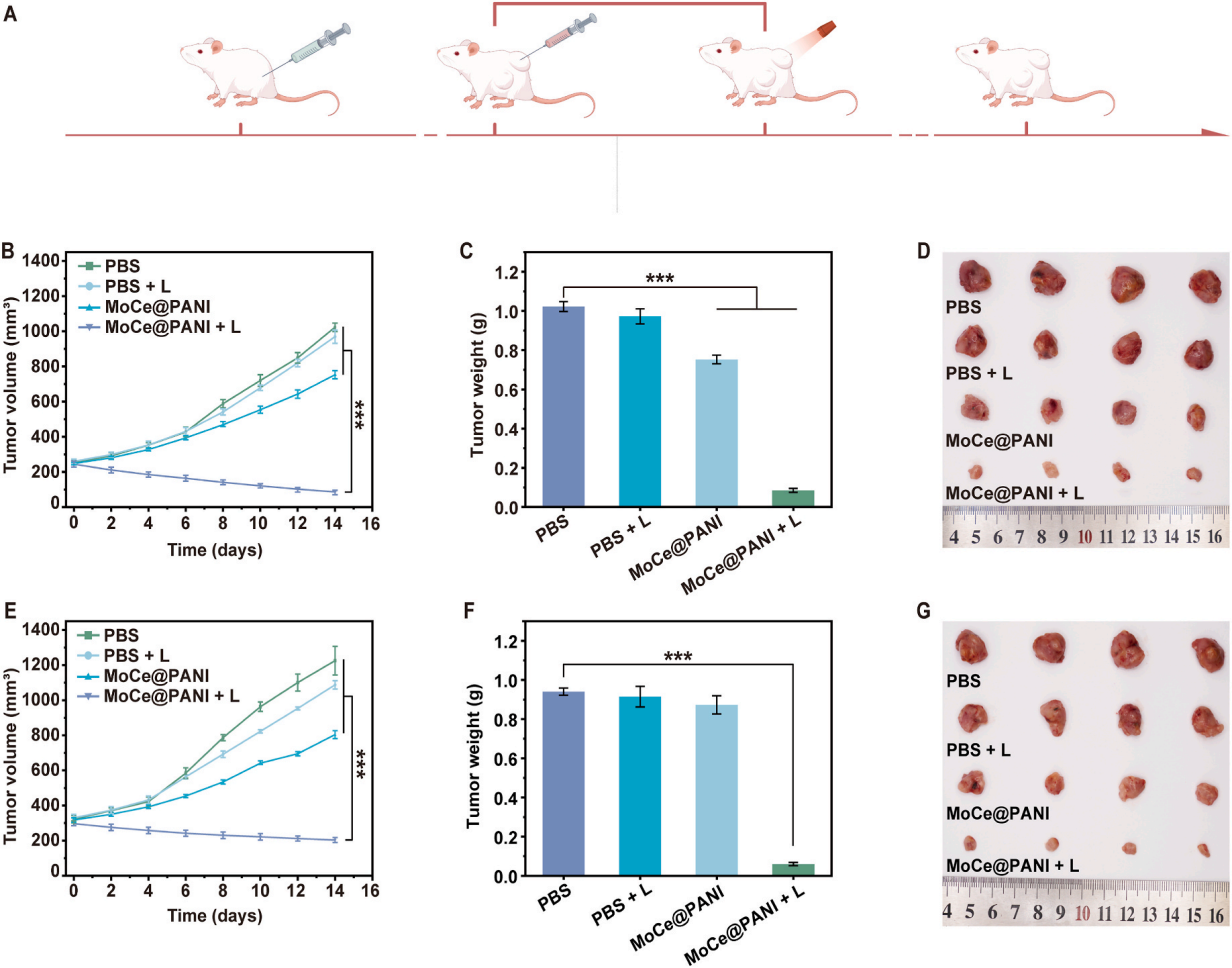
Abscopal effect of MoCe@PANI NPs-mediated photothermal therapy in a bilateral 4T1 tumor model. (A) Schematic illustration of the experimental timeline and treatment protocol. (B–D) Primary tumors (irradiated side): (B) Tumor growth curves, (C) *Ex vivo* tumor weights, and (D) Representative photographs. (E–G) Distant tumors (non-irradiated side): (E) Tumor growth curves, (F) *Ex vivo* tumor weights, and (G) Representative photographs. Data are presented as mean ± SD (n = 4). *p < 0.05, **p < 0.01, ***p < 0.001.

To investigate the potential of local synergistic therapy to induce a systemic antitumor immune response, a bilateral 4T1 tumor model was established (Fig. 7A). The bilateral model employed intratumoral injection into the right-sided tumor (primary tumor), in contrast to the intravenous injection used in the unilateral therapeutic study (Fig. 6). This change in administration route was deliberate: intratumoral injection maximizes local drug retention at the primary treatment site while minimizing systemic distribution to the contralateral (distant) tumor, thereby ensuring the distant tumor remains genuinely untreated—an essential prerequisite for unbiased evaluation of the abscopal effect. Following intratumoral injection of MoCe@PANI NPs, the primary tumor was irradiated with a 1064 nm laser at 24 h post-injection, a time point selected based on our *in vivo* PA imaging data demonstrating that MoCe@PANI NP accumulation in tumors peaked at 24 h (Fig. 5F). The left-sided tumor (distant tumor) was left entirely untreated to evaluate the abscopal effect.

For the primary tumors on the irradiated side, the combination of MoCe@PANI NPs and laser (MoCe@PANI + L) exerted potent tumor inhibition. Tumor growth curves showed that the MoCe@PANI + L group exhibited significantly delayed progression compared with the PBS, MoCe@PANI alone, and PBS + L groups (Fig. 7B). Correspondingly, the excised tumor weights on day 14 were dramatically reduced in the combined treatment group (Fig. 7C). Representative photographs of the primary tumors further confirmed the smallest tumor sizes in the MoCe@PANI + L group (Fig. 7D).

**Fig. 8:**
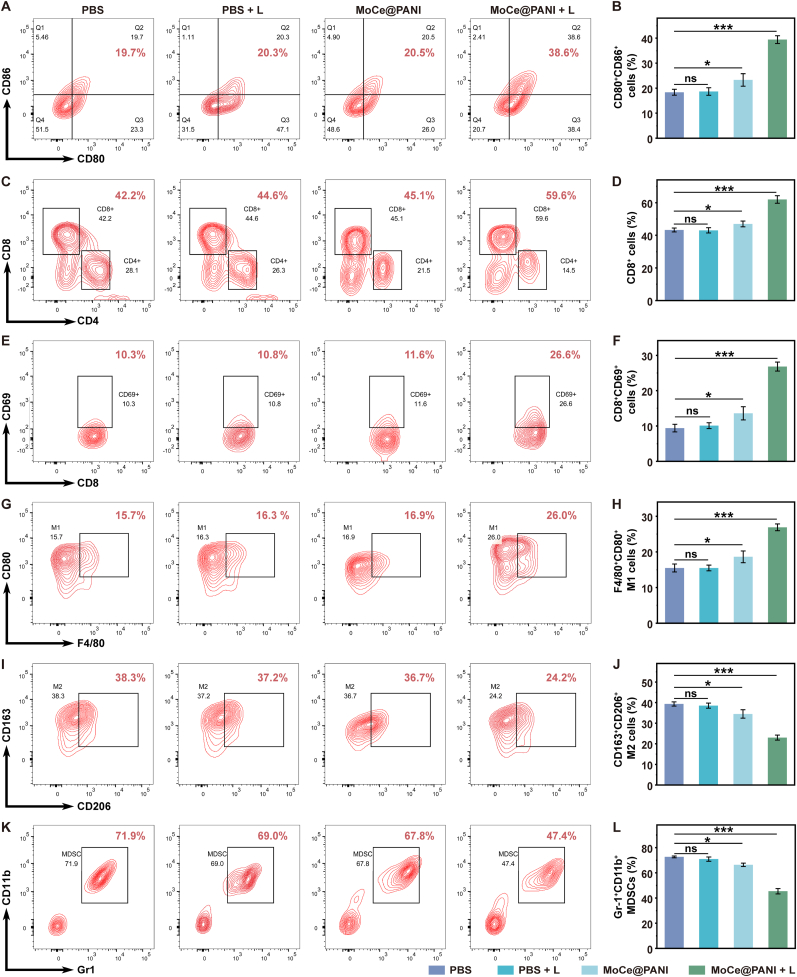
*In vivo* immune responses induced by MoCe@PANI NPs-mediated synergistic therapy. Flow cytometry analysis of (A, B) DC maturation (CD80^+^CD86^+^), (C, D) CD8^+^ and CD4^+^ T cells in tumors, (E, F) CD69 expression on CD8^+^ T cells, (G, H) M1 macrophages (F4/80^+^CD80^+^), (I, J) M2 macrophages (CD163^+^CD206^+^), and (K, L) MDSCs (CD11b^+^Gr-1^+^). Data are presented as mean ± SD (n = 3). *p < 0.05, **p < 0.01, ***p < 0.001; ns, not significant.

Remarkably, a strong abscopal effect was observed in the distant tumors that received no direct intervention. While distant tumors in the control groups grew aggressively, those in the MoCe@PANI + L group displayed substantially suppressed growth (Fig. 7E) and markedly lower tumor weights (Fig. 7F). Photographs of excised distant tumors visually demonstrated that the combined treatment applied to the primary site effectively restrained the progression of untreated distant tumors (Fig. 7G). Collectively, these results indicate that MoCe@PANI NPs-mediated synergistic therapy triggers a systemic immune response capable of inhibiting distant tumor growth. Notably, the abscopal effect observed here depends on the immunostimulatory nature of the therapy itself—ICD induction leading to DC maturation, CD8^+^ T cell activation, and TME remodeling as demonstrated below (Fig. 8)—rather than on the administration route, confirming that the intratumoral route in this model serves solely to ensure the experimental rigor of the abscopal evaluation.

### 3.8 In vivo immunomodulatory effects and TME remodeling

To elucidate the immunological mechanisms underlying the observed abscopal effect, we performed a comprehensive flow cytometry analysis of immune cells within the tumor microenvironment (TME). The synergistic therapy was hypothesized to reverse the immunosuppressive TME into an immunostimulatory one, thereby facilitating systemic antitumor immunity.

First, we evaluated the maturation of dendritic cells (DCs), which are pivotal for antigen presentation and T cell priming. As shown in Fig. 8A and B, the MoCe@PANI + Laser group exhibited a significantly higher percentage of mature DCs (CD80^+^CD86^+^) compared to the control groups. This indicates that the combination therapy effectively promoted DC maturation, likely due to the release of tumor-associated antigens and DAMPs during immunogenic cell death (ICD). Consequently, this enhanced antigen presentation capability led to the robust recruitment and activation of T lymphocytes. Flow cytometry quantification revealed a marked increase in the infiltration of both cytotoxic CD8^+^ T cells and helper CD4^+^ T cells in the combined treatment group (Fig. 8C and D), providing the essential effector cells for tumor elimination. Crucially, to confirm the functional status of these infiltrated T cells, we analyzed the expression of CD69, an early activation marker. As depicted in Fig. 8E and F, the proportion of CD69^+^ cells within the CD8^+^ T cell population (CD8^+^CD69^+^) was significantly upregulated in the MoCe@PANI + Laser group. This finding confirms that the therapy not only increases the number of CD8^+^ T cells but also ensures their functional activation rather than exhaustion.

Following T cell activation, we investigated the subsequent remodeling of the myeloid compartment, specifically the polarization of tumor-associated macrophages (TAMs). Typically, M2-like macrophages promote tumor growth and suppress immunity, whereas M1-like macrophages exhibit pro-inflammatory and antitumor activities. Our results showed a distinct shift in macrophage phenotype upon treatment. The proportion of pro-inflammatory M1-like macrophages (F4/80^+^CD80^+^) increased substantially (Fig. 8G and H), while the population of immunosuppressive M2-like macrophages (CD163^+^CD206^+^) decreased significantly (Fig. 8I and J). This repolarization from an M2 to an M1 phenotype, likely driven by IFN-γ secreted from the activated CD8^+^ T cells, suggests that the therapy successfully remodeled the TME from an immunosuppressive state to an immunostimulatory one.

Finally, we assessed the levels of myeloid-derived suppressor cells (MDSCs), a heterogeneous population known for suppressing antitumor immune responses. As illustrated in Fig. 8K and L, the abundance of MDSCs (CD11b^+^Gr-1^+^) was markedly reduced in the MoCe@PANI + Laser group. The depletion of MDSCs further alleviated the immunosuppressive burden within the TME, creating a favorable environment for effective T cell-mediated killing.

Collectively, these findings demonstrate that the synergistic combination of CDT and NIR-II PTT not only directly kills tumor cells but also orchestrates a potent antitumor immune response. By promoting DC maturation, recruiting and activating CD8^+^ T cells, repolarizing macrophages to the M1 phenotype, and depleting MDSCs, this strategy effectively remodels the cold TME into a hot, immunologically active environment. This comprehensive immune activation provides a mechanistic explanation for the inhibition of distant tumors observed in the bilateral model, highlighting the potential of MoCe@PANI NPs as a promising platform for cancer immunotherapy.

## 4 Conclusions

In conclusion, this study presents MoCe@PANI NPs that orchestrate a synergistic therapeutic strategy by combining NIR-II photothermal therapy (PTT) with GSH-depletion-augmented chemodynamic therapy (CDT). Our findings demonstrate that MoCe@PANI NPs function as dual-active nano-agents capable of catalyzing the conversion of endogenous H_2_O_2_ into highly toxic hydroxyl radicals while simultaneously exhausting intracellular GSH reserves. This cascade disrupts redox homeostasis, promotes lipid peroxidation, suppresses GPX4 expression, and ultimately drives tumor cells into ferroptosis via mitochondrial collapse. The superior NIR-II photothermal performance of MoCe@PANI NPs serves a dual purpose: it induces direct thermal ablation and thermally accelerates the Fenton-like reaction kinetics, creating a self-reinforcing cycle of oxidative damage. Beyond direct tumor killing, this synergistic regimen triggers robust immunogenic cell death (ICD), which promotes dendritic cell maturation, primes cytotoxic T lymphocytes, and repolarizes macrophages from the pro-tumor M2 phenotype to the antitumor M1 phenotype, thereby remodeling the immunosuppressive tumor microenvironment into an immune-active state. Collectively, the MoCe@PANI system exemplifies a powerful “all-in-one” theranostic platform bridging material science and immunology. This work offers a compelling blueprint for developing next-generation nanomedicines capable of effectively suppressing primary tumors, inhibiting distant metastases, and reducing recurrence risk through comprehensive immune activation.

## Ethics approval and consent to participate

Our research involve experimentation on animals. All animal work was compliant with the Guangdong provincial people's hospital (approval number: KY2023-901). And we confirmed authors' compliance with all relevant ethical regulations.

## CRediT authorship contribution statement

**Yulin Kuang:** Data curation, Formal analysis, Investigation, Writing – original draft. **Chen Lu:** Formal analysis, Investigation, Writing – original draft. **Bolan Yu:** Formal analysis, Investigation, Writing – original draft. **Lie Li:** Formal analysis, Investigation, Writing – original draft. **Fei Xiao:** Formal analysis, Investigation, Writing – original draft. **Xuyuan Chen:** Formal analysis, Investigation, Writing – original draft. **Junwei Wang:** Formal analysis, Investigation, Writing – original draft. **Liming Lei:** Formal analysis, Investigation, Writing – original draft. **Yijin Wu:** Formal analysis, Investigation, Writing – original draft. **Xinjian Yan:** Formal analysis, Investigation, Writing – original draft. **Shuoji Zhu:** Conceptualization, Data curation, Formal analysis, Investigation, Project administration, Writing – original draft, Writing – review & editing. **Min Wu:** Conceptualization, Data curation, Formal analysis, Investigation, Project administration, Writing – original draft, Writing – review & editing. **Nanbo Liu:** Conceptualization, Data curation, Formal analysis, Investigation, Project administration, Writing – original draft, Writing – review & editing. **Ruiyuan Liu:** Conceptualization, Data curation, Formal analysis, Investigation, Project administration, Writing – original draft, Writing – review & editing. **Ping Zhu:** Conceptualization, Funding acquisition, Project administration, Supervision, Writing – original draft, Writing – review & editing.

## Declaration of competing interest

The authors declare that they have no known competing financial interests or personal relationships that could have appeared to influence the work reported in this paper.
