# Supplementary material for: Cerium molybdate-doped polyaniline nanoplatform for GSH-depletion-enhanced chemodynamic/NIR-II photothermal synergistic therapy via ferroptosis and immune activation
Source: Bioact Mater. 2026 Aug 12;67:371–86. doi: 10.1016/j.bioactmat.2026.06.035 (PMC13487302; doi:10.1016/j.bioactmat.2026.06.035)
Supplement: Supplementary file 1 [file mmc1.doc]

**Supplementary Information**

**Cerium Molybdate-Doped Polyaniline Nanoplatform for GSH-Depletion-Enhanced Chemodynamic/NIR-II Photothermal Synergistic Therapy via Ferroptosis and Immune Activation**

**Materials**

All chemical reagents and solvents were of analytical grade and used directly without further purification unless otherwise specified.

**Synthesis of control materials**

Free PANI (without Mo/Ce) and free MoCe (without PANI) were synthesized following the same procedure as for MoCe@PANI NPs, except that (i) for free PANI, the metal precursors (ammonium molybdate and cerium(IV) nitrate) were omitted and replaced with an equal volume of pre‑cooled deionized water; and (ii) for free MoCe, the aniline monomer was omitted. MoCe@PANI NPs with different metal loadings (25 mg and 200 mg) were prepared by adjusting the amounts of ammonium molybdate and cerium(IV) nitrate to 25 mg and 200 mg (each), respectively, while keeping all other parameters unchanged.

**Calculation of Photothermal Conversion Efficiency**

The photothermal conversion efficiency ( *η* ) was calculated according to Roper's method. The temperature change of the MoCe@PANI NPs aqueous dispersion (1 mL, 400 μg/mL) was recorded under continuous 1064 nm laser irradiation (1.0 W/cm²) until a steady state was reached, followed by natural cooling.

*η* = *hA* ( *T*_max_−*T*_sur_ )−*Q*_dis_ / *I* ( 1−10^−^*^Aλ^* )

Where *h* is the heat transfer coefficient, *A* is the surface area of the container, *T*_max_ is the equilibrium temperature (50.8 °C), *T*_sur_ is the ambient temperature (28.8 °C), *Q*_dis_ represents heat dissipation from the solvent/cell (measured using pure water), *I* is the laser power (1.0 W), and *Aλ* is the absorbance at 1064 nm (0.613).

The heat transfer coefficient (*hA*) was derived from the cooling period time constant (*τ*_s_):

*hA = m*_d_ *c*_d_ */ τ*_s_

Where *m*_d_ (1.0 g) and *c*_d_ (4.2 J/(g·°C)) are the mass and heat capacity of water, respectively. *τ*_s_ was determined to be 519.3 s from the linear fitting of the cooling curve (−lnθ vs. time). Based on these parameters, *η* was calculated to be 48.2%.

**Table S1. ICP-MS quantitative analysis data of Mo and Ce in MoCe@PANI NPs**

| **Tested element** | **Mo-1** | **Mo-2** | **Mo-3** | **Ce-1** | **Ce-2** | **Ce-3** |
| --- | --- | --- | --- | --- | --- | --- |
| **Actual sample volume (mL)** | 1 | 1 | 1 | 1 | 1 | 1 |
| **Constant volume *V₀* (mL)** | 10 | 10 | 10 | 10 | 10 | 10 |
| **Element concentration in test solution *C₀* (μg/L)** | 0.902 | 0.935 | 0.932 | 1.217 | 1.25 | 1.275 |
| **Dilution factor *f*** | 10 | 10 | 10 | 10 | 10 | 10 |
| **Element concentration in digestion solution *C₁* (μg/L)** | 9.02 | 9.35 | 9.32 | 12.17 | 12.50 | 12.75 |
| **Element content in sample *Cₓ* (μg/kg)** | 90.2 | 93.5 | 93.2 | 121.7 | 125 | 127.5 |
| **Element content in sample *W* (%)** | 9.02×10^-6^ | 9.35×10^-6^ | 9.32×10^-6^ | 1.217×10^-5^ | 1.250×10^-5^ | 1.275×10^-5^ |

Fig. S1. Representative TEM images of MoCe@PANI NPs prepared with different metal precursor amounts: (A) 25 mg, (B) 100 mg (original formulation), and (C) 200 mg. Scale bar: 500 nm.

Fig. S2. Size distribution of MoCe@PANI NPs stored at RT for 14 days.

Fig. S3. TEM of MoCe@PANI NPs stored in PBS (pH = 7.4) for 7 days.

**Fig. S4.** UV-Vis-NIR absorption spectra of free PANI, free MoCe, and MoCe@PANI with different metal loadings (25, 100, 200 mg). Concentration: 400 μg/mL in water. Dashed line: 1064 nm.

Fig. S5. Infrared (IR) thermal images of MoCe@PANI NPs (0, 25, 50, 100, 200, 400 μg/mL) under 1064 nm laser irradiation (1.0 W/cm^2^) at different time points.

Fig. S6. Infrared (IR) thermal images of MoCe@PANI NPs under 1064 nm laser irradiation (0, 0.5, 1.0, 1.5, 2.0 W/cm²) at various time points.

Fig. S7. MoCe@PANI NPs deplete GSH at different time points (DTNB assay).

**Fig. S8.** In vitro cytotoxicity of free PANI, free MoCe, and MoCe@PANI with different metal loadings (25, 100, 200 mg) against 4T1 cells without or with 1064 nm laser irradiation (1.0 W/cm², 5 min). Nanoparticle concentration: 400 μg/mL. Cell viability was measured by CCK-8 assay.

**Fig. S9.** TMB oxidation assay for ·OH generation by free PANI, free MoCe, and MoCe@PANI with different metal loadings (25, 100, 200 mg) with or without 1064 nm laser irradiation (1.0 W/cm², 5 min). Absorbance at 652 nm was recorded.

**Fig. S10**. Biodistribution of MoCe@PANI NPs in major organs and tumor at 6, 12, 24, and 48 h post-intravenous injection measured by ICP-MS. (A) Ce content (%ID/g). (B) Mo content (%ID/g).

**Fig. S11**. Body weight changes of mice after injection with MoCe@PANI NPs with different concentrations.

**Fig. S12**. Hematoxylin and eosin (H&E) staining of the main organs after different treatments.
